# Type 2 Diabetes Mellitus in Inflammatory Bowel Disease Patients: A Case–Control Study Through a Long Follow-Up Period

**DOI:** 10.3390/jcm14010143

**Published:** 2024-12-30

**Authors:** Benedetta Zaccardi, Angelo Armandi, Gian Paolo Caviglia, Fabio Broglio, Marta Vernero, Michelle Bombonato, Beatrice Giannone, Guglielmo Beccuti, Davide Giuseppe Ribaldone

**Affiliations:** Department of Medical Sciences, University of Turin, 10126 Turin, Italy; benedettazaccardi@gmail.com (B.Z.); angelo.armandi@unito.it (A.A.); gianpaolo.caviglia@unito.it (G.P.C.); fabio.broglio@unito.it (F.B.); marta.vernero@unito.it (M.V.); michelle.bombonato@unito.it (M.B.); beatricegiannone@unito.it (B.G.); davidegiuseppe.ribaldone@unito.it (D.G.R.)

**Keywords:** anti-TNF-alpha, corticosteroids, Crohn’s disease, IBD, liver damage, liver steatosis, severe IBD, type 2 diabetes mellitus, ulcerative colitis

## Abstract

**Background/Objectives:** The characterization of patients with inflammatory bowel disease (IBD) and type 2 diabetes mellitus (T2DM) as a new group has not been well detailed. This study aimed to evaluate the impact of T2DM on IBD progression and analyze the prevalence of steatotic liver disease and liver damage in these patients. **Methods:** Through a retrospective case–control study, we compared severe IBD occurrence in patients with both IBD-T2DM (cases) versus those with IBD alone (controls). Among 1047 medical records, 79 IBD-T2DM patients were selected and compared to 308 controls in a 1:4 ratio. Severe IBD was defined by variables such as surgery, target therapy, corticosteroid use, and hospitalization. Liver damage was assessed using Fib-4 (>1.3), and hepatic steatosis was evaluated by imaging. **Results:** There was no significant difference in severe disease rates (59.5% vs. 59.7%; *p* = 0.97). IBD-T2DM patients had higher rates of hepatic steatosis (62.9% vs. 27.2%; *p* < 0.0001) and liver damage (55.4% vs. 26.6%; *p* < 0.0001). IBD-T2DM patients used more corticosteroids (*p* < 0.0001) and fewer anti-TNF-alpha drugs (*p* = 0.007). The median age at diagnosis was higher in IBD-T2DM patients (48 vs. 32; *p* < 0.0001). In Crohn’s disease, 24.3% of IBD-T2DM patients had exclusive colonic involvement compared to 5% in the IBD-only group (*p* = 0.003). **Conclusions:** T2DM was not associated with worse IBD progression, but was linked to increased liver steatosis and damage. Differences such as age of onset, colonic involvement, and liver damage suggest that IBD-T2DM patients could configure a special population worthy of further studies.

## 1. Introduction

Both inflammatory bowel disease (IBD) and type 2 diabetes mellitus (T2DM) are chronic, multifactorial diseases influenced by environmental factors acting on genetic predisposition.

A diet rich in fats, particularly animal fats, and polyunsaturated fatty acids has been associated with an increased incidence of Crohn’s disease (CD) and ulcerative colitis (UC), as well as type 2 diabetes mellitus (T2DM), due to its influence of increasing the systemic inflammatory status [1]. Certain dietary components can indeed affect the structure and function of the microbiota, with downstream effects on immune activity [2]. A factor implicated in both diseases is intestinal dysbiosis; both diseases share an increase in the presence of microbial species with pathogenic potential, such as *Ruminococcus gnavus*, which utilizes mucin and dietary glycans and produces bacteriocins and adhesins. These compounds elicit a pro-inflammatory host response and modulate host metabolism of bile acids and tryptophan metabolic pathways [3,4]. Conversely, there is a decrease in *Akkermansia muciniphila*, a bacterium that plays a beneficial role in maintaining intestinal mucosal integrity and reducing the production of pro-inflammatory substances [5].

The growing interest in the management of comorbidities, within an increasingly elderly population, has prompted several research groups to investigate the relationship between these diseases.

In a cohort study conducted in Denmark on 6,028,844 people, a higher number of diabetic subjects was observed in the cohort of IBD patients compared to the general population, resulting in a 50% higher risk of IBD patients developing T2DM [6]. In another cohort study conducted in South Korea, an increased relative risk (RR) of 1.09 was observed in IBD patients for developing T2DM: 1.41 in CD patients and 1.04 in UC patients [7]. Additionally, in a cohort study conducted in China, involving 454,804 individuals, CD patients showed an increased risk of T2DM compared to the general population, as well as UC patients [8].

Corticosteroid therapy is one of the main assumptions underlying the link between IBD and T2DM, as steroids are currently a first-line therapy to induce remission in IBD exacerbations [9,10]. The effect of systemic corticosteroids (but also, partially, topical corticosteroids) of raising blood glucose has been widely demonstrated [11].

In mouse models, hyperglycemia has been observed to worsen the clinical outcomes of IBD activity, presumably acting on the integrity of the colonic epithelial barrier [12]. However, the role of T2DM in the course of IBD in clinical trials is controversial. For example, in Uwagbale et al.’s study, patients with IBD and T2DM were found to have a lower risk of IBD-related complications and a lower risk of undergoing IBD-related surgery, compared to patients with isolated IBD; on the other hand, an increase in septic complications was highlighted [13]. An increase in septic complications was also described by Kumar et al., together with an increase in hospitalizations, IBD-related complications, and disease flare-ups, despite a non-significant difference in the incidence of IBD-related surgeries [14]. Fuschillo et al. conducted a meta-analysis, where it emerged that T2DM in patients with IBD did not worsen the course of the disease in terms of IBD-related complications, IBD-related surgery, and mortality, despite representing an independent risk factor for IBD-related hospitalizations and sepsis [15].

The characterization of the new category of patients with IBD and T2DM has not been well detailed yet: studies on the effects of diabetes on inflammatory disease have yielded conflicting results. Among the possible explanations, the heterogeneity of IBD clinical presentations stands out, along with the variability in the criteria for endpoint attribution and the description of results [15]. Therefore, to assess how T2DM might influence the course of intestinal disease, we selected the definition of severe IBD as the primary objective of our study, independent of the increased comorbidity risk associated with T2DM.

Furthermore, both IBD and T2DM patients are at an elevated risk for liver disease compared to the general population [16,17,18], yet the extent of these complications in patients with both conditions has been poorly investigated. Consequently, as a secondary endpoint, we examined the prevalence of steatotic liver disease (SLD) and liver damage in patients with IBD and T2DM.

## 2. Materials and Methods

We conducted a retrospective case–control study to compare patients with both IBD and T2DM to patients who had IBD without T2DM. We reviewed the medical records of patients followed at the IBD Clinical Center of the ‘San Giovanni Antica Sede’ Hospital, AOU Città della Salute e della Scienza in Turin, Italy.

### 2.1. Inclusion and Exclusion Criteria

Inclusion in cases:

- Minimum follow-up period of 1 year;
- Confirmed diagnosis of IBD according to the 2019 ECCO-ESGAR guidelines [19];
- History of T2DM at the time of their last visit.

Exclusion in cases:

- Inability to determine severe disease according to a composite outcome that considers IBD manifestations and complications described by Yarur et al. (see Section 2.3) [20].

Inclusion in controls:

- Minimum follow-up period of 1 year;
- Confirmed diagnosis of IBD according to the 2019 ECCO-ESGAR guidelines [19].

Exclusion in controls:

- Inability to determine severe disease according to a composite outcome that considers IBD manifestations and complications described by Yarur et al. (see Section 2.3) [20];
- Medical history of T2DM or iatrogenic diabetes;
- Past/current therapy with hypoglycemic drugs.

**Figure 1:**
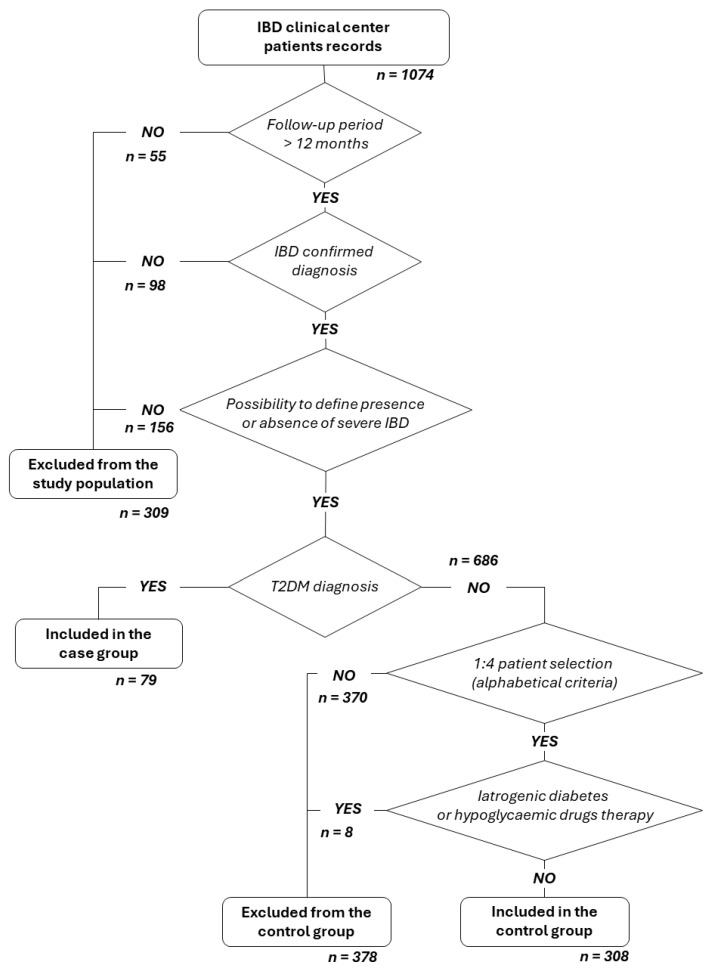
Patient qualification flow chart.

Figure 1 presents the patient qualification flow chart.

The control group was randomly selected from selected IBD patients (in alphabetical order) at an approximate ratio of 1:4 between cases and controls. 

### 2.2. Type and Source of Data

In Table 1 type and source of data are listed.

### 2.3. Endpoint Definition

The primary endpoint of this study was to determine the prevalence of severe IBD in IBD-T2DM patients compared to a control group of IBD patients without T2DM. For this purpose, we used a composite outcome that considers IBD manifestations and complications described by Yarur et al. in a systematic review aimed at establishing predictors of aggressive IBD [20]. Thus, patients were considered to have severe IBD if at least one of the following conditions occurred during the entire follow-up period:

Need for IBD-related surgery; need for immunosuppressive drugs/biologics/small molecules as maintenance therapy; more than two courses of systemic corticosteroids during the entire duration of follow-up; need for hospitalization for flare-ups or complications after initial diagnosis; at least one of the following: presence of persistent diarrhea for more than 12 months with nocturnal awakenings or urgency, intestinal stricture with symptoms.

Moreover, we chose the presence of liver damage and the presence of hepatic steatosis as secondary endpoints.

We defined liver damage through the use of the FIB4 score, calculated as age in years × AST in U/L/platelets in ×10⁹/L × √ALT in U/L [21]; in this study, a result higher than 1.3 determined the presence of liver damage. This endpoint was evaluated only in patients whose records reported AST, ALT, and platelet count values at least once. Where multiple measurements were reported, the most recent ones were taken into consideration.

We took into consideration the presence of SLD if steatosis was described in medical history, and if examinations by abdominal ultrasound or FibroScan^®^ were suggestive of it. Patients who had never undergone ultrasound/Fibroscan were not evaluated. The absence of steatosis was defined for those patients who underwent at least one of the previous imaging investigations and in whom hepatic steatosis was not described in their medical history, the liver was normal, or the CAP values were normal.

### 2.4. Statistical Analysis

Continuous variables were reported as mean (standard deviation (SD)) or median (interquartile range (IQR)) based on the distribution of values. Normality was verified using the D’Agostino–Pearson test. Categorical variables were reported as numbers and percentages.

Comparison between independent categorical variables was performed using the Chi-square test. Comparison between continuous variables was performed using the independent samples t-test or the Mann–Whitney test based on the distribution of values.

The association between variables was tested using the proportional hazard model or Cox regression; therefore, the strength of the association was reported as a hazard ratio (HR) with a 95% confidence interval (95% CI).

All statistical analyses were performed using MedCalc^®^ v. 20.104 (MedCalc Software Ltd., Ostend, Belgium), and a *p* value ≤ 0.05 was considered statistically significant.

## 3. Results

We analyzed 1047 medical records, from which we selected 79 patients with IBD-T2DM (confirmed diagnosis of both diseases), comparing the selected population with 308 patients with IBD alone, randomly chosen in a 1:4 ratio. Baseline characteristics of the entire study population (n = 387) are reported in Table 2.

In Table 3, a comparison of baseline characteristics in cases and controls is shown.

**Figure 2:**
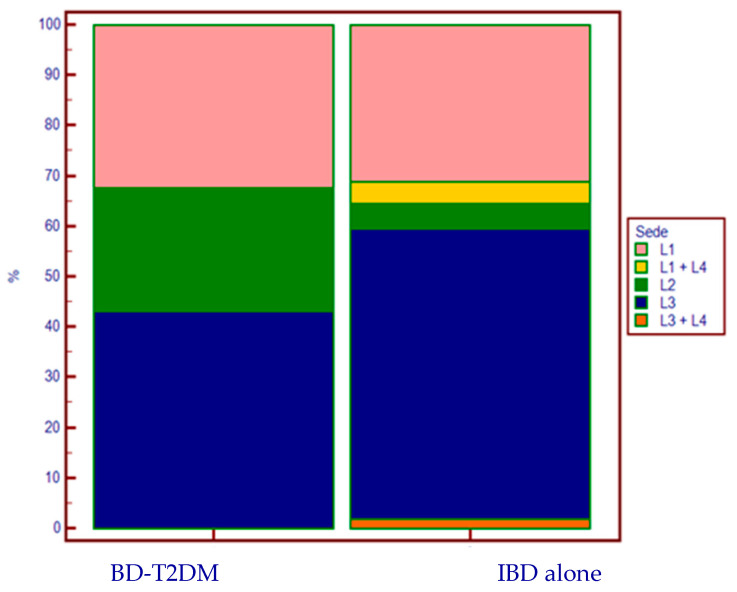
CD’s localization according to the presence (or absence) of T2DM.

We observed a statistically significant difference in the disease localization of CD in the two groups (Figure 2).

**Figure 3:**
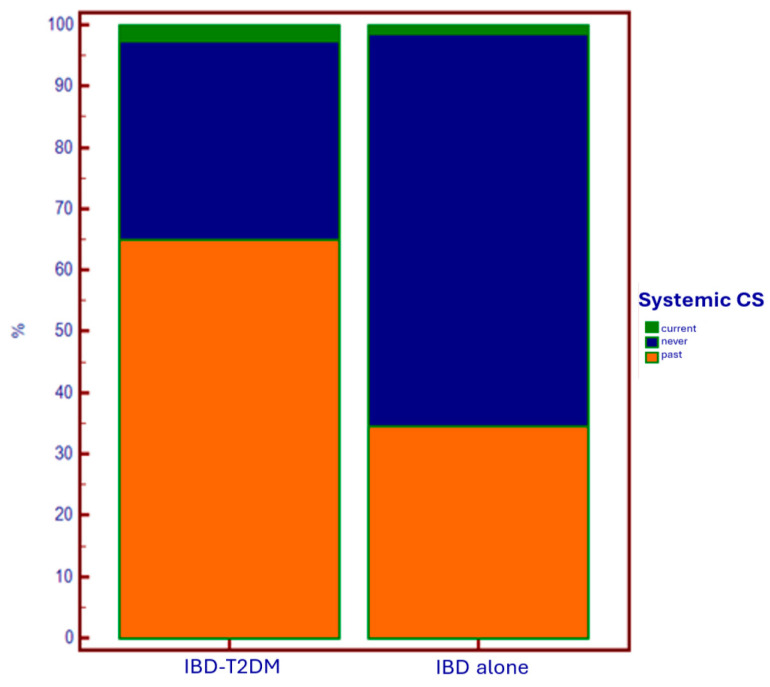
Use of systemic corticosteroids based on the presence of T2DM.

As regards the specific therapy for intestinal disease (Table 3), there was a greater current and past use of systemic corticosteroids (CSs) in the T2DM group compared to the group with IBD alone (*p* < 0.0001) (Figure 3).

**Figure 4:**
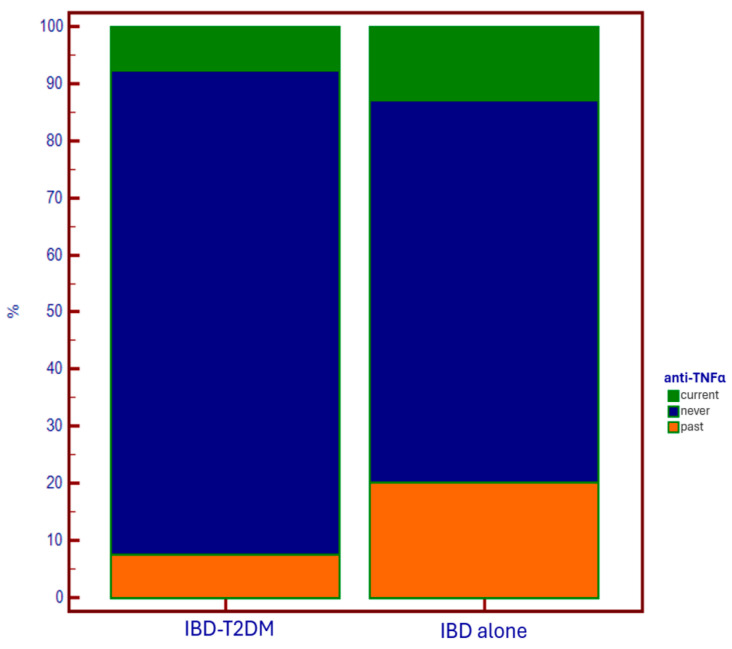
Use of anti-TNF-alpha drugs based on the presence of T2DM.

Lower use of anti-TNF-alpha drugs was observed in the T2DM group, both in current therapy and in the past compared to controls (*p* = 0.007) (Figure 4).

### 3.1. T2DM Patients

We were able to exactly determine when the diagnosis of T2DM occurred in 35 out of 79 patients. The average age at diagnosis was 56.2 years (±12.6). Among them, 25 patients were diagnosed after IBD diagnosis, with a mean IBD-T2DM latency of 18.0 years (±11.1). There were also 10 patients who developed diabetes before bowel disease, with a median latency of −3.0 years (IQR: −8.0 to −1.0).

As regards specific therapies for diabetes, the absolute and relative frequencies by pharmacological class found in the study group are reported in Supplementary Table S1.

### 3.2. Metabolic Parameters

For all the preset metabolic parameters, except for alcohol intake, we observed a significant difference between the two groups (see Table 4).

### 3.3. Severe IBD

In Table 5, the comparison of baseline characteristics based on the presence or absence of severe IBD is reported.

We performed a univariate analysis (in Table 6) for those variables which, using the Chi-square test and the T-test, showed a statistically significant association for the outcome “severe IBD”.

The multivariate analysis regarding predictors of severe IBD is reported in Table 7.

### 3.4. Liver Damage

Table 8 describes the comparison of baseline characteristics based on the presence or absence of liver damage defined by a Fib 4 score > 1.3.

Then, we performed a univariate analysis for the variables that, in the Chi-square test and the T-test, showed a statistically significant association with the outcome Fib-4 > 1.3. As reported in Table 9, three dichotomous variables and one continuous variable were identified as significant in increasing the risk of liver damage during the duration of follow-up.

In Table 10, the multivariate analysis of predictors for Fib-4 > 1.3 is described.

## 4. Discussion

Median age at IBD diagnosis was significantly higher in cases (48.0), while in controls (32.0; *p* < 0.0001), the median age fell within the range reported in epidemiology for this pathology (25–35 years) [22]. This result has not been described in the literature to date.

In the sub-analysis in patients with CD, there was a notable difference in the disease location of activity between cases and controls: 24.3% of the CD-DMT2 group had a disease localized exclusively at the colonic level (L2), compared to 5% in the group without T2DM (*p* = 0.003). Furthermore, no upper-intestinal localization (L4) was found in any case, whereas it was present in 10 patients in the control group (6.2%). This distribution does not reflect the percentages described in the literature [22,23], and could suggest another distinct characteristic of this special subpopulation.

Regarding specific therapy for IBD, in diabetic patients, we observed a wider (current and especially past) administration of systemic corticosteroids, and a lower administration of anti-TNF-alpha drugs. The first data could support an iatrogenic explanation of the epidemiological relationship between IBD and T2DM. On the other hand, in patients using anti-TNF-alpha as maintenance therapy for disease control, there is a lower need for steroids. This results in a reduced need to undergo steroid courses due to disease flare-ups, which has a favorable impact on reducing the risk of developing diabetes associated with steroid administration.

The reason why anti-TNF-alpha agents are underutilized in patients with diabetes is likely due to the increased fragility of these patients, both because of the immune system deficiency that makes them more susceptible to infections (to which anti-TNF-alpha agents could further expose them), and due to the older age of this population, which is associated with the presence of multiple comorbidities, including those in which anti-TNF-alpha agents are contraindicated (e.g., heart failure). In this context, the use of anti-integrin and anti-IL12/23 agents would certainly be appropriate options, thanks to the lower immunosuppressive effect and the better safety profile [24,25]. Conversely, the use of anti-JAK molecules would not be a good choice for these patients, considering the EMA alert on their administration in populations with increased metabolic/thrombotic risk, and suggesting that they be considered only in the absence of other alternatives [26]. Anyway, it is clear how in IBD-T2DM patients, it is fundamental to identify the best therapeutic strategy that allows for a reduction in steroid administration, through optimal disease control. In fact, the usage of steroids in patients who already have a diagnosis of diabetes is implicated in the occurrence of diabetes decompensation and, consequently, the development of multiple complications [27].

Among patients with IBD and T2DM, 67.1% were hypertensive, while 59.7% were dyslipidemic (vs. 18.6% and 21.2% in controls). However, these percentages were comparable to those found in diabetics without intestinal comorbidity [28].

Regarding the presence of severe IBD, as the primary endpoint, no significant difference emerged from the comparison between the IBD + T2DM group and the controls, with a prevalence of 59.5 and 59.7%, respectively. These data partly differ from the literature, where an increase in IBD-related hospitalizations (included in the composite endpoint) in IBD-T2DM patients is reported [14,15], despite very discordant data among studies [13,15]. Then, our study confirmed a lack of increased need for IBD surgery in IBD-T2DM patients [13,14,15].

In our cohort, 69.3% of patients with severe IBD were diagnosed with CD, whereas only 41.7% of patients who did not have severe IBD were diagnosed with CD (*p* < 0.0001). However, in the univariate analysis, which accounts for the different follow-up lengths for each patient, the presence of CD was not associated with an increased risk of a severe IBD course (HR 1.25; 95% CI 0.94–1.65).

In our study, being naïve to SGLT2 inhibitors, although significant in the χ^2^ test (27.7% vs. 12.5%; *p* = 0.0383), was not protective against severe disease in the univariate analysis. Instead, being naïve to GLP-1 ra was significantly protective for severe IBD in the univariate analysis, but this significance was not confirmed in the multivariate analysis. Currently, in the literature, there are no clinical studies on the prescription of SGLT2i in the specific population of IBD-DMT2. As regards GLP-1 ra, one study assumed that these drugs could improve the course of IBD [29], but a similar result was not observed in our study.

The only significant predictor for severe disease in uni- and multivariable analysis was the age at last follow-up: increasing age decreases the risk of severe IBD (HR: 0.98; 95% CI: 0.97 to 0.99; *p* < 0.0001). This finding may be explained by the fact that IBD in elderly patients has a more indolent presentation [30], with symptoms and complications that do not fit our definition of serious disease.

Regarding secondary endpoints, the comparison between the IBD-T2DM group and controls revealed a more than doubled prevalence of hepatic steatosis in cases (62.9% vs. 27.2%; *p* < 0.0001). However, the prevalence of steatosis in IBD-T2DM patients did not differ from the prevalence reported in the general diabetic population [31]; thus, we presume that the association of the two pathologies does not provide a multiplicative risk factor for steatosis.

The assessment of liver damage, using the Fib-4 score, differs significantly between cases and controls (55.4% vs. 26.6%; *p* < 0.0001). However, it is likely that the difference in average age (higher in diabetic patients of 19.2y) has negatively influenced score parameters (ALT, AST, and no. of platelets) [32,33]. Evaluating the predictors of Fib-4 score > 1.3, DMT2 increased the risk for liver damage (HR: 1.57) with a trend toward statistical significance in the univariate analysis (95% CI 0.99 to 2.51; *p* = 0.06).

In the sub-analysis evaluating the characteristics of the population regarding the presence of liver damage, the association of hypertension (HR: 1.61; *p* = 0.047) and dyslipidemia (HR: 1.68; *p* = 0.03) was statistically significant in the univariate analysis. This aligns with the numerous studies describing metabolic syndrome and its effects on the liver [16,31,34]. Moreover, in the univariate analysis, we observed that being naïve to thiopurine constituted another risk factor for liver damage (HR: 1.81; *p* = 0.02), even though the contribution of the covariates hypertension, dyslipidemia, and being naïve to thiopurines was not significant in the multivariate analysis. The contribution of being naïve to anti-TNF-alpha drugs was not significant.

In the multivariable analysis on predictors of liver damage, age was the only covariate resulting in a significant increase in risk (HR 1.03; 95% CI 1.01–1.05; *p* = 0.01). This result, although relevant from a statistical point of view, raises doubts about the clinical usefulness of a non-specific predictor such as Fib-4 in the older population due to the presence of age in the numerator of the score, as already highlighted by van Kleef et al. [33].

No association between liver damage and alcohol consumption was observed, although alcohol is one of the most important causes of liver fibrosis and an increase in hepatic cytolysis indices. However, it is possible that this result was conditioned by the data collection method, since the definition of alcohol consumption included the occasional drink, so it was not possible to define the alcohol units consumed.

## 5. Chapter Limitation and Future Directions

Regarding the criticisms of our study, one of the main limitations is its retrospective nature, which does not allow us to establish a causal link between the associations found. Furthermore, the analysis of metabolic characteristics between cases and controls revealed several variables that could potentially introduce biases, since conditions such as T2DM, hepatic steatosis, hypertension, dyslipidemia, and obesity share many common causative factors. However, the effect of these potential biases was mitigated by multivariate analyses. Another limitation derives from the monocentric design of this study.

It would certainly be useful for future studies to conduct prospective research with well-defined endpoints in order to reduce the biases typical of retrospective studies.

## 6. Conclusions

In our study, the presence of T2DM was not associated with a worse clinical course of IBD.

In patients with IBD, T2DM has been associated with a higher presence of steatotic liver disease and liver damage, but the IBD-T2DM association is not a multiplicative risk factor for these outcomes.

Regarding specific therapies for IBD, in patients with T2DM, we observed a greater administration (current and especially past) of systemic corticosteroids in clinical practice and a lower usage of anti-TNF-alpha drugs.

The differences in age at onset of the pathology, the greater prevalence of colonic involvement, and the greater presence of liver damage suggest that patients with IBD and T2DM could represent a special population worthy of further studies.

## Figures and Tables

**Table 1:** Type and source of data.

Data Collected for All Subjects	Details
Personal data	Age, sex
Physiological history	Weight, height, smoking habits, alcohol consumption
Intestinal disease details	Type, location, date of diagnosis, presence of perianal disease, indicators of severe disease, current and past therapy
Blood tests	AST, ALT, platelet count
Metabolic data	Hypertension, dyslipidemia
**Additional data collected for cases**	**Details**
Details of diabetes	Date of diagnosis, type, presence of complications, current and past therapy
T2DM complications *	Atherosclerotic cardiovascular disease (ASCVD), diabetic nephropathy, diabetic retinopathy, diabetic neuropathy

* Patients with at least one of these complications were classified as having complicated T2DM.

**Table 2:** Baseline characteristics of the study population.

Variables	
Age at last check-up (years, mean ± SD)	57.7 ± 17.5
Follow-up duration (years, median (IQR))	15 (8–27)
Age of IBD diagnosis (years, median (IQR))	35 (25–50)
Type of IBD	
CD (n, %)	37 (46.8%)
IBD-U (n, %)	6 (7.6%)
UC (n, %)	36 (45.6%)
UC localization	
E1 (n, %)	14 (11.2%)
E2 (n, %)	59 (47.2%)
E3 (n, %)	52 (41.6%)
CD localization	
L1 (n, %)	62 (31.3%)
L1 + L4 (n, %)	7 (3.5%)
L2 (n, %)	17 (8.6%)
L3 (n, %)	109 (55.1%)
L3 + L4 (n, %)	3 (1.5%)
Smoking	
Current (n, %)	117 (30.3%)
Ex-smoker (n, %)	110 (28.5%)
Never (n, %)	29 (36.7%)
Systemic corticosteroids	
Current (n, %)	6 (1.7%)
Never (n, %)	202 (57.1%)
Past (n, %)	146 (41.2%)
Thiopurine	
Current (n, %)	22 (5.8%)
Never (n, %)	249 (65.4%)
Past (n, %)	110 (28.9%)
MTX	
Current (n, %)	4 (1.2%)
Never (n, %)	327 (95.3%)
Past (n, %)	12 (3.5%)
Anti-TNF-alpha	
Current (n, %)	44 (11.7%)
Never (n, %)	266 (70.7%)
Past (n, %)	66 (17.6%)
Anti-integrin	
Current (n, %)	22 (5.9%)
Never (n, %)	335 (89.6%)
Past (n, %)	17 (4.5%)
Anti-IL12/IL23	
Current (n, %)	25 (6.7%)
Never (n, %)	342 (91.7%)
Past (n, %)	6 (1.6%)
Anti-JAK	
Current (n, %)	1 (0.3%)
Never (n, %)	272 (99.2%)
Past (n, %)	2 (0.5%)

IQR = interquartile range; SD = standard deviation; MTX = methotrexate; current = patient currently on therapy; never = patient naïve to that drug; past = the patient has taken the drug at least once; anti-TNF-alpha = anti-tumor necrosis factor alpha; anti-IL12/23 = anti-interleukin 12/23; anti-JAK = Janus kinase inhibitors.

**Table 3:** Comparison of baseline characteristics between the two groups.

Variables	IBD + T2DM (N = 79)	Controls (N = 308)	
Age at last check-up (years, mean ± SD)	71.0 ±10.8	54.2 ±17.3	*p* < 0.0001
Females	19 (24.1%)	148 (48.1%)	*p* = 0.0001
Age at IBD diagnosis (years, median [IQR])	48.0 [36.0–62.0]	32.0 [24.5–45.0]	*p* < 0.0001
Follow-up duration (years, median [IQR])	14.0 [7.0–25.0]	20.0 [10.5–33.0]	*p* = 0.0001
Type of IBD (n, %)			*p* = 0.07
CD	37 (46.8%)	188 (61.0%)	
IBD-U	6 (7.6%)	15 (4.9%)	
UC	36 (45.6%)	105 (34.1%)	
UC localization (n, %)			*p* = 0.70
E1	4 (11.1%)	10 (11.2%)	
E2	19 (52.8%)	40 (44.9%)	
E3	13 (36.1%)	39 (43.8%)	
CD localization (n, %)			*p* = 0.002
L1	12 (32.4%)	50 (31.1%)	
L1 + L4	0	7 (4.3%)	
L2	9 (24.3%)	8 (5.0%)	
L3	16 (43.2%)	93 (57.8%)	
L3 + L4	0	3 (1.9%)	
Smoking (n, %)			*p* < 0.0001
Current	11 (13.9%)	106 (34.5%)	
Ex-smoker	39 (49.4%)	71 (23.1%)	
Never	29 (36.7%)	130 (42.3%)	
Severe IBD (n, %)	47 (59.5%)	184 (59.7%)	*p* = 0.97
Systemic corticosteroids (n, %)			*p* < 0.0001
Current	2 (2.6%)	4 (1.4%)	
Never	25 (32.5%)	177 (63.9%)	
Past	50 (64.9%)	96 (34.7%)	
Topical corticosteroids (n, %)			*p* = 0.22
Current	15 (23.4%)	33 (14.7%)	
Never	35 (54.5%)	128 (57.1%)	
Past	14 (21.9%)	63 (28.1%)	
Thiopurines (n, %)			*p* = 0.87
Current	5 (6.3%)	17 (5.6%)	
Never	53 (67.1%)	196 (64.9%)	
Past	21 (26.6%)	89 (29.5%)	
MTX (n, %)			*p* = 0.98
Current	1 (1.3%)	3 (1.1%)	
Never	74 (94.9%)	253 (95.5%)	
Past	3 (3.8%)	9 (3.4%)	
Anti-TNF-alpha (n, %)			*p* = 0.007
Current	6 (7.6%)	38 (12.8%)	
Never	67 (84.8%)	199 (67.0%)	
Past	6 (7.6%)	60 (20.2%)	
Anti-integrins (n, %)			*p* = 0.30
Current	7 (8.9%)	15 (5.1%)	
Never	70 (88.6%)	265 (89.8%)	
Past	2 (2.5%)	15 (5.1%)	
Anti-IL12/IL23 (n, %)			*p* = 0.23
Current	2 (2.5%)	23 (7.8%)	
Never	76 (96.2%)	266 (90.5%)	
Past	1 (1.3%)	5 (1.7%)	
Anti-JAK (n, %)			*p* = 0.67
Current	0	1 (0.3%)	
Never	79 (100.0%)	293 (99.0%)	
Past	0	2 (0.7%)	

IQR = interquartile range; SD = standard deviation; MTX = methotrexate; current = patient currently on therapy; never = patient naïve to that drug; past = the patient has taken the drug at least once; anti-TNF-alpha = anti-tumor necrosis factor alpha; anti-IL12/23 = anti-interleukin 12/23; anti-JAK = Janus kinase inhibitors.

**Table 4:** Metabolic parameters in participants.

Metabolic Variables	IBD + T2DM	Controls	*p* Value
Hypertension	51 (67.1%)	49 (18.6%)	<0.0001
Dyslipidemia	46 (59.7%)	53 (21.2%)	<0.0001
Alcohol	29 (54.7%)	64 (37.4%)	=0.03
Hepatic steatosis	44 (62.9%)	34 (27.2%)	<0.0001
Fib-4 > 1.3	31 (55.4%)	42 (26.6%)	<0.0001
BMI (median)	26.6	23.4	<0.0001

BMI = body mass index.

**Table 5:** Characteristics regarding the presence or absence of severe IBD.

	Severe IBD (n = 231)	No Severe IBD (n = 156)	*p* Value
Age at last follow-up visit (years, mean ± SD)	59.2 ± 16.7	55.4 ± 18.5	*p*^a^ = 0.04
Females	95 (39.8%)	75 (48.1%)	*p* = 0.10
Age at IBD diagnosis (years, median [IQR])	35.0 [25.0–49.0]	35.0 [26.0–50.0]	*p*^b^ = 0.56
Median follow-up duration (years, median [IQR])	18.0 [10.0–28.0]	10.0 [6.0–21.5]	*p*^b^ < 0.0001
Type of IBD			
CD	160 (69.3%)	65 (41.7%)	
IBD-U	13 (5.6%)	8 (5.1%)	
UC	58 (25.1%)	83 (53.2%)	*p* < 0.0001
Smoking			*p* = 0.93
Current	69 (29.9%)	48 (31.0%)	
Ex-smoker	65 (28.1%)	45 (29.0%)	
Never	97 (42.0%)	62 (40.0%)	
Diabetes			*p* = 0.97
Y	47 (59.5%)	32 (40.5%)	
N	184 (59.7%)	124 (40.3%)	
Metformin			*p* = 0.87
Current	25 (54.3%)	19 (59.4%)	
Never	17 (37.0%)	10 (31.2%)	
Past	4 (8.7%)	3 (9.4%)	
Insulin			*p* = 0.84
Current	10 (21.3%)	8 (25.0%)	
Never	31 (66.0%)	19 (59.4%)	
Past	6 (12.7%)	5 (15.6%)	
GLP1-ra			*p* = 0.03
Current	4 (8.5%)	10 (31.2%)	
Never	40 (85.1%)	20 (62.5%)	
Past	3 (6.4%)	2 (6.3%)	
DPP4i			*p* = 0.49
Current	5 (10.6%)	5 (15.6%)	
Never	41 (87.2%)	25 (78.1%)	
Past	1 (2.1%)	2 (6.2%)	
SGLT2-i			
Current	13 (27.7%)	4 (12.5%)	
Never	34 (72.3%)	25 (78.1%)	
Past	0	3 (9.4%)	*p* = 0.04
Glitazones			*p* = 0.4517
Current	3 (6.4%)	3 (9.4%)	
Never	42 (89.4%)	29 (90.6%)	
Past	2 (4.2%)	0	
Sulfonylureas			*p* = 0.95
Current	2 (4.3%)	1 (3.1%)	
Never	37 (78.7%)	25 (78.1%)	
Past	8 (17.0%)	6 (18.8%)	
Glinides			*p* = 0.71
Current	2 (4.3%)	2 (6.3%)	
Never	44 (95.7%)	30 (93.7%)	
Acarbose			*p* = 0.50
Current	1 (2.1%)	0	
Never	45 (95.7%)	32 (100%)	
Past	1 (2.1%)	0	

IQR = interquartile range; SD = standard deviation; ^a^ T-Test; ^b^ Mann-Whitney Test; current = patient currently on therapy; never = patient naïve to that drug; past = the patient has taken the drug at least once; GLP1-ra = glucagon-like peptide-1 receptor agonists; DPP4i = dipeptidyl peptidase-4 inhibitors; SGLT2-i = sodium–glucose co-transporter 2 inhibitors.

**Table 6:** Univariate analysis based on the presence of severe IBD for the duration of follow-up.

	HR	95% CI	*p* Value
IBD = “CD”	1.25	0.94–1.65	0.13
GLP-1 ra = “Never”	0.67	0.47–0.94	0.02
SGLT2i = “Never”	0.74	0.51–1.07	0.11
Age at last follow-up	0.98	0.97–0.99	<0.0001

GLP1-ra = glucagon-like peptide-1 receptor agonists; never = patient naïve to that drug; SGLT2-i = sodium–glucose co-transporter 2 inhibitors.

**Table 7:** Multivariate analysis based on the presence of severe IBD for the duration of follow-up.

	HR	95% CI	*p* Value
Age	0.98	0.97–0.99	<0.0001
GLP-1 ra = “Never”	0.85	0.58–1.23	0.37

GLP1-ra = glucagon-like peptide-1 receptor agonists; never = patient naïve to that drug.

**Table 8:** Characteristics based on the presence or absence of Fib 4 > 1.3.

Variables	Fib-4 > 1.3	Fib-4 <= 1.3	
Average age	68.9 ± 11.0	49.7 ± 15.1	*p* < 0.0001
Diabetes	31 (42.5%)	25 (17.7%)	*p* = 0.0001
Complicated diabetes	13 (41.9%)	7 (29.1%)	*p* = 0.33
Severe IBD	47 (64.4%)	92 (65.2%)	*p* = 0.90
Hypertension	39 (53.4%)	30 (21.7%)	*p* < 0.0001
Dyslipidemia	30 (43.5%)	31 (23.0%)	*p* = 0.003
Alcohol	23 (50.0%)	41 (41.4%)	*p* = 0.33
Systemic corticosteroids			*p* = 0.90
Current	1 (1.5%)	1 (0.8%)	
Never	37 (54.4%)	71 (54.6%)	
Past	30 (44.1%)	58 (44.6%)	
Thiopurines			*p* = 0.02
Current	4 (5.5%)	9 (6.3%)	
Never	53 (72.6%)	76 (53.9%)	
Past	16 (21.9%)	56 (39.7%)	
MTX			*p* = 0.17
Current	3 (4.1%)	1 (0.7%)	
Never	66 (90.4%)	135 (95.7%)	
Past	4 (5.5%)	5 (3.5%)	
Anti-TNF-alpha			*p* = 0.03
Current	9 (12.5%)	25 (18.0%)	
Never	53 (73.6%)	77 (55.4%)	
Past	10 (13.9%)	37 (26.6%)	
Anti-integrins			*p* = 0.43
Current	4 (5.6%)	15 (10.7%)	
Never	64 (87.6%)	117 (83.6%)	
Past	5 (6.8%)	8 (5.7%)	
Anti-IL12/IL23			*p* = 0.78
Current	7 (9.7%)	12 (8.6%)	
Never	64 (88.9%)	124 (88.6%)	
Past	1 (1.4%)	4 (2.8%)	
Anti-JAK			*p* = 0.45
Current	0	1 (0.7%)	
Never	73 (100%)	138 (97.9%)	
Past	0	2 (1.4%)	

MTX = methotrexate; current = patient currently on therapy; never = patient naïve to that drug; past = the patient has taken the drug at least once; anti-TNF-alpha = anti-tumor necrosis factor alpha; anti-IL12/23 = anti-interleukin 12/23; anti-JAK = Janus kinase inhibitors.

**Table 9:** Univariate analysis of predictors of Fib-4 > 1.3 for duration of follow-up.

	HR	95% CI	*p* Value
Diabetes	1.57	0.99 to 2.51	0.06
Hypertension	1.61	1.01 to 2.58	0.047
Dyslipidemia	1.68	1.04 to 2.70	0.03
Thiopurines (never)	1.81	1.08 to 3.04	0.02
Anti-TNF-alpha (never)	1.33	0.79 to 2.24	0.28
Age at last follow-up	1.04	1.02 to 1.06	0.0002

Never = patient naïve to that drug; anti-TNF-alpha = anti-tumor necrosis factor alpha.

**Table 10:** Multivariate analysis of predictors of Fib-4 > 1.3.

	HR	95% CI	*p* Value
Hypertension	1.12	0.67 to 1.86	0.67
Dyslipidemia	1.38	0.83 to 2.27	0.21
Thiopurines (never)	1.50	0.89 to 2.55	0.13
Age at last follow-up	1.03	1.01 to 1.05	0.008

## Data Availability

The data presented in this study are available on request from the corresponding author.

## Supplementary Materials

The following supporting information can be downloaded at https://www.mdpi.com/article/10.3390/jcm14010143/s1: Table S1: Antidiabetic therapy in the IBD + T2DM group.
