# Supplementary material for: Type 2 Diabetes Mellitus in Inflammatory Bowel Disease Patients: A Case–Control Study Through a Long Follow-Up Period
Source: J Clin Med. 2024 Dec 30;14(1):143. doi: 10.3390/jcm14010143 (PMC11721770; doi:10.3390/jcm14010143)
Supplement: Supplementary file 1 [file jcm-14-00143-s001.zip › jcm-3372689-supplementary.pdf]

Supplementary Table S1. Antidiabetic therapy in the IBD + T2DM group.

Antidiabetic drug	n	%
Metformin		
current	44	56.4%
never	27	34.6%
past	7	9.0%
Insulin		
current	18	22.8%
never	50	63.3%
past	11	13.9%
GLP1-ra		
current	14	17.7%
never	60	75.9%
past	5	6.3%
DPP4i		
current	10	12.7%
never	66	83.5%
past	3	3.8%
SGLT2-i		
current	17	21.5%
never	59	74.7%
past	3	3.8%
Glitazones		
current	6	7.6%
never	71	89.9%
past	2	2.5%
Sulfonylureas		
current	3	3.8%
never	62	78.5%
past	14	17.7%
Glinides		
current	4	5.1%
never	74	94.9%
Acarbose		
current	1	1.3%
never	77	97.5%
past	1	1.3%

current = patient currently on therapy; never = patient naïve to that drug; past = the patient has taken the drug at least once; GLP1-ra = glucagon-like peptide-1 receptor agonists; DPP4i = dipeptidyl peptidase-4 inhibitors; SGLT2-i = sodium–glucose co-transporter 2 inhibitors.
